# Epidemiology and infectious complications of pediatric burns in French Guiana: a 6-year retrospective study

**DOI:** 10.3389/fpubh.2026.1844435

**Published:** 2026-06-16

**Authors:** Jeanne Chambon, Olivier Corseri, Anicet Sika, Patrice Guemaleu, Narcisse Elenga

**Affiliations:** Centre Hospitalier de Cayenne, Cayenne, French Guiana

**Keywords:** burn injury, epidemiology, French Guiana, infection, pediatric

## Abstract

**Background:**

Pediatric burns represent an important cause of hospitalization and morbidity among children worldwide. The aim of this study was to describe the epidemiological, clinical, and microbiological characteristics of children hospitalized for burns, with a focus on infectious complications and factors associated with invasive infection in French Guiana. Secondary objectives included the identification of factors associated with infectious complications.

**Methods:**

A retrospective study was conducted at Cayenne Hospital between January 2017 and December 2022. Invasive infection was operationally defined as sepsis or septic shock according to pediatric SIRS-based criteria associated with suspected or documented infection.

**Results:**

A total of 165 patients were included in the study. The mean age at admission was 45.5 months, and 94 patients (57%) were male. The most frequent injuries were small-surface burns (<10% total body surface area), superficial partial-thickness burns caused by thermal mechanisms, mainly scalding. Involvement of high-risk areas such as the face or perineum was observed in 42% of patients. The mean length of hospital stay was 9.8 days, and 10% of patients required medical evacuation to a specialized burn center. Wound colonization was common, with 87% of skin swab cultures returning positive results; the most frequently isolated bacterium was methicillin-sensitive *Staphylococcus aureus* (MSSA). Eighteen of 79 blood cultures (22.8%) were positive., most commonly identifying MSSA followed by Gram-negative bacteria. Forty-eight patients (29%) received antibiotic therapy for a mean duration of 7.3 days. Urinary catheterization was performed in 23 patients, including 16 in the non-sepsis group and 7 in the sepsis group. Overall, 7.9% of patients developed signs of invasive infection, defined as sepsis or septic shock. Univariate analysis identified younger age (21 months vs. 4 years) and a larger burned body surface area (15% vs. 8%) as factors associated with invasive infection.

**Conclusion:**

Thermal partial-thickness burns were the most common type of burn injury among hospitalized children included in this cohort and predominantly affected children under 5 years of age.of age. Invasive infections remained relatively uncommon in this single-center cohort. Younger age, larger burned body surface area, residence in remote areas, and the use of invasive devices were associated with invasive infection.

## Introduction

Burn injuries represent a major public health problem worldwide and, according to the World Health Organization (WHO), are responsible for approximately 180,000 deaths each year (1). Children are particularly vulnerable, and burns represent the fifth leading cause of non-fatal trauma in this population (2).

The incidence of burns is closely associated with socioeconomic conditions. Most burns occur in the home environment and are strongly linked to poverty, overcrowding, lack of safety measures, and domestic activities such as cooking or caring for young children.

French Guiana is one of the poorest regions of France and has one of the fastest-growing and youngest populations in the country (3). Given these demographic characteristics, burn injuries appear to represent a frequent reason for pediatric consultation (4).

Infectious complications are common among burn patients and represent one of the leading causes of morbidity and mortality (5). In children, this risk is even greater due to their immature immune system and thinner skin barrier, making infections the leading cause of death among severely burned pediatric patients.

To date, data on children hospitalized for burn injuries in French Guiana remain scarce. Improved knowledge of the epidemiological and clinical characteristics of these patients could contribute to a better understanding of patient profiles and lead to improved clinical management protocols.

The primary objective of this study was to describe the epidemiological, clinical, and microbiological characteristics of children hospitalized for burns at Cayenne Hospital Center. Secondary objectives included the identification of factors associated with infectious complications.

## Methods

### Study design and setting

A retrospective descriptive study was conducted over a 6-year period, from January 2017 to December 2022, at Cayenne Hospital Center, French Guiana.

Cayenne Hospital Center is an 800-bed public hospital with missions in healthcare, teaching, and research. It provides both referral and local care through a network of 16 healthcare centers located throughout the territory. Cayenne Hospital Center functions both as the main referral hospital for pediatric burn patients in French Guiana and as a transfer point to specialized burn centers in mainland France for the most severe cases. The hospital also coordinates care through a network of decentralized healthcare centers distributed across remote areas of the territory. This organizational structure may influence both the epidemiological profile of hospitalized patients and variables such as length of stay and infection management. Burn patients were managed in a general pediatric ward or in the pediatric intensive care unit (fPICU of our beds) according to burn severity, hemodynamic status, respiratory involvement, and the need for invasive monitoring or organ support. The multidisciplinary management involved pediatricians, intensivists, surgeons, anesthesiologists, and specialized nursing staff.

### Patient selection

The inclusion criteria were all pediatric patients aged between 1 month and 16 years who were hospitalized in the pediatric department—either in the medical ward, surgical ward, or pediatric intensive care unit—for burn management between January 2017 and December 2022.

The exclusion criterion was patients managed for burn injuries on an outpatient basis.

Eligible patients were identified through the hospital medical information department database.

### Data collection

Medical records of the included patients were reviewed, and the following variables were collected.

Sociodemographic variables

- Age
- Sex
- City of origin

Regarding the place of residence, patients were categorized into two groups:

1. Patients living in Cayenne and its surrounding municipalities (including Rémire-Montjoly, Matoury, Macouria, and Kourou)
2. Patients from remote or isolated communes, who required an initial medical transfer to reach Cayenne Hospital Center.

### Clinical variables

The following clinical variables were collected:

- Time to first medical contact
- Burn mechanism (classified as projection/scald, immersion, contact, flame, electrical, or chemical burn)
- Total burned body surface area (TBSA)
- Burn depth. Burn depth was categorized as first-degree, superficial second-degree, deep second-degree, or third-degree burn according to standard clinical assessment based on skin appearance, capillary refill, blistering, pain sensitivity, and tissue involvement.
- Burn location
- Presence of lesions in high-risk areas (extremities, genitals/perineum, inhalation injury, periocular region, neck, or circular burns)
- Length of hospital stay
- Need for medical evacuation to a specialized burn center

### Infectious and microbiological variables

The following infection-related variables were analyzed:

- Occurrence of wound colonization
- Occurrence of sepsis or septic shock
- Microorganisms identified in wound cultures
- Microorganisms identified in blood cultures
- Antibiotic therapy administered
- Duration of antibiotic treatment (in days)

Wound cultures consisted of superficial skin swab cultures performed according to routine clinical practice in patients with fever, elevated inflammatory markers, larger burns, delayed wound healing, or clinical suspicion of infection.

### Definitions of infection

For the purpose of this study, invasive infection was operationally defined as the occurrence of sepsis or septic shock.

Systemic Inflammatory Response Syndrome (SIRS) was defined as the presence of at least two of the following criteria (4):

- Core temperature > 38.5 °C or < 36 °C
- Tachycardia or bradycardia
- Respiratory rate > 2 standard deviations above normal for age or need for mechanical ventilation
- Leukopenia or leukocytosis
- Systolic blood pressure < 5th percentile for age

Although Sepsis-3 definitions were introduced during the study period, pediatric-specific validation remains limited, particularly in retrospective burn cohorts. Therefore, pediatric SIRS-based definitions derived from the 2012 Surviving Sepsis Campaign guidelines were used for consistency with previous pediatric burn literature.

Sepsis was defined as SIRS associated with a suspected or documented infection, such as (4):

- High bacterial load in wound colonization. Wound cultures consisted of superficial skin swab cultures performed according to routine clinical practice.
- Purulent or suppurative lesions
- Positive blood culture.

Septic shock was defined as sepsis associated with persistent hemodynamic instability after initial fluid resuscitation (40 mL/kg) or by the combination of the following criteria (4):

- Sepsis
- Need for vasopressor therapy to maintain mean arterial pressure above the lower limit for age
- Lactate level > 2 mmol/L despite adequate fluid resuscitation.

Wound colonization alone was not considered invasive infection and microbiological findings were interpreted together with clinical criteria.

Detailed surgical management data were inconsistently documented in the medical records and could not be reliably analyzed.

### Therapeutic variables

Therapeutic data were also collected, including:

- Administration of antibiotic therapy
- Duration of treatment (in days)

The main criteria used for transfer to specialized burn centers, included extensive burns, inhalation injury, and need for advanced surgical management. Transferred patients were included only for the duration of hospitalization at Cayenne Hospital Center and that no follow-up data from referral centers were available.

### Statistical analysis

Data were collected and recorded in an Excel spreadsheet designed specifically for the study. Continuous quantitative variables were described using measures of central tendency (mean and median), while categorical variables were expressed as frequencies and percentages. Categorical variables were compared using Chi-square or Fisher’s exact tests as appropriate. Continuous variables were compared using Student’s t-test or Mann–Whitney U test depending on data distribution. Due to the limited number of invasive infection events, multivariable analysis was not considered sufficiently robust and only univariate associations are presented. A *p*-value < 0.05 was considered statistically significant. The infection-related variables were analyzed as binary outcomes rather than time-dependent events. Patients transferred to specialized burn centers were included only for the duration of hospitalization at Cayenne Hospital Center. Follow-up data after transfer were not available.

### Ethics

In accordance with French and European regulations, including Regulation (EU) 2016/679 (General Data Protection Regulation), and considering the retrospective nature of the study (research not involving human subjects – *Recherche n’impliquant pas la personne humaine*, RnIPH; Articles 44-1 and 65-2 of the French Data Protection Act and Article L1110-12 of the French Public Health Code), submission to an ethics committee was not required.

The study was conducted in accordance with the Declaration of Helsinki. All data were retrospectively collected and pseudonymized from patients’ medical records by the principal investigator. As part of this internal research protocol, patients were informed about the study through individual information notices, and any expressed objection to participation was respected when applicable. The study was reviewed and authorized according to institutional procedures for retrospective non-interventional studies at Cayenne Hospital Center and declared to the CNIL (Commission Nationale de l’Informatique et des Libertés) in accordance with current French data protection regulations.

## Results

### General characteristics

Between January 2017 and December 2022, 185 patients were initially identified for the study. Twenty patients were subsequently excluded: nine due to an incorrect primary diagnosis, seven who were managed on an outpatient basis, three due to incomplete medical records, and one because the hospitalization occurred outside the study period. A total of 165 patients were included in the final analysis.

Sociodemographic and clinical characteristics are presented in Table 1. Most patients were male (57%) and lived in Cayenne or its surrounding areas. The mean age at admission was 45.5 months, with a median age of 21 months. Most patients (85%) had their first medical contact within the first 24 h after the burn injury.

**Table 1:** Patient characteristics.

Variable	Value
Number of patients	165
Demographic characteristics	
Age at admission, months	45.5 ± 46.4
Median age, months (IQR)	21 (13–76)
Male sex	94 (57%)
Residence in Cayenne area	109 (66%)
Residence in remote communes	56 (34%)
Clinical characteristics	
Time to first medical contact <24 h	141 (85%)
24–72 h	13 (7.9%)
>72 h	11 (6.7%)
TBSA burned (%)	8.8 ± 8.85
Median TBSA (IQR)	6.4 (3.6–10)
Length of hospital stay (days)	9.8 ± 11
Median length of stay (IQR)	7 (3–12)
Burn characteristics	
*Burn mechanism*	
Scald (projection)	112 (68%)
Flame	20 (12%)
Contact	14 (8.5%)
Electrical	10 (6.1%)
Immersion	7 (4.2%)
Chemical	2 (1.2%)
*Burn depth*	
1st degree	2 (1.2%)
Superficial 2nd degree	110 (67%)
Deep 2nd degree	41 (25%)
3rd degree	12 (7.3%)
*Total burned body surface area (TBSA)*	
<10%	122 (74%)
10–20%	25 (15%)
20–40%	15 (9.1%)
>40%	3 (1.8%)
*High-risk burn areas*	
Any high-risk area	70 (42%)
Inhalation injury	2 (1.2%)
Neck	7 (4.2%)
Perineum/genitals	18 (11%)
Extremities	35 (21%)
Circular burns	15 (9.1%)
Eyes	11 (6.7%)
*Medical evacuation to burn center*	
Yes	17 (10%)
No	148 (90%)

TBSA, Total burned body surface area.

The majority of burns involved a small total body surface area (TBSA <10%), were superficial second-degree burns, and were mainly caused by scald injuries due to liquid projection. However, involvement of high-risk anatomical areas (face, extremities, perineum, neck, eyes, inhalation injury, or circular burns) was observed in 42% of patients. Urinary catheterization was performed in 23 patients, including 16 in the non-sepsis group and 7 in the sepsis group.

**Figure 1:**
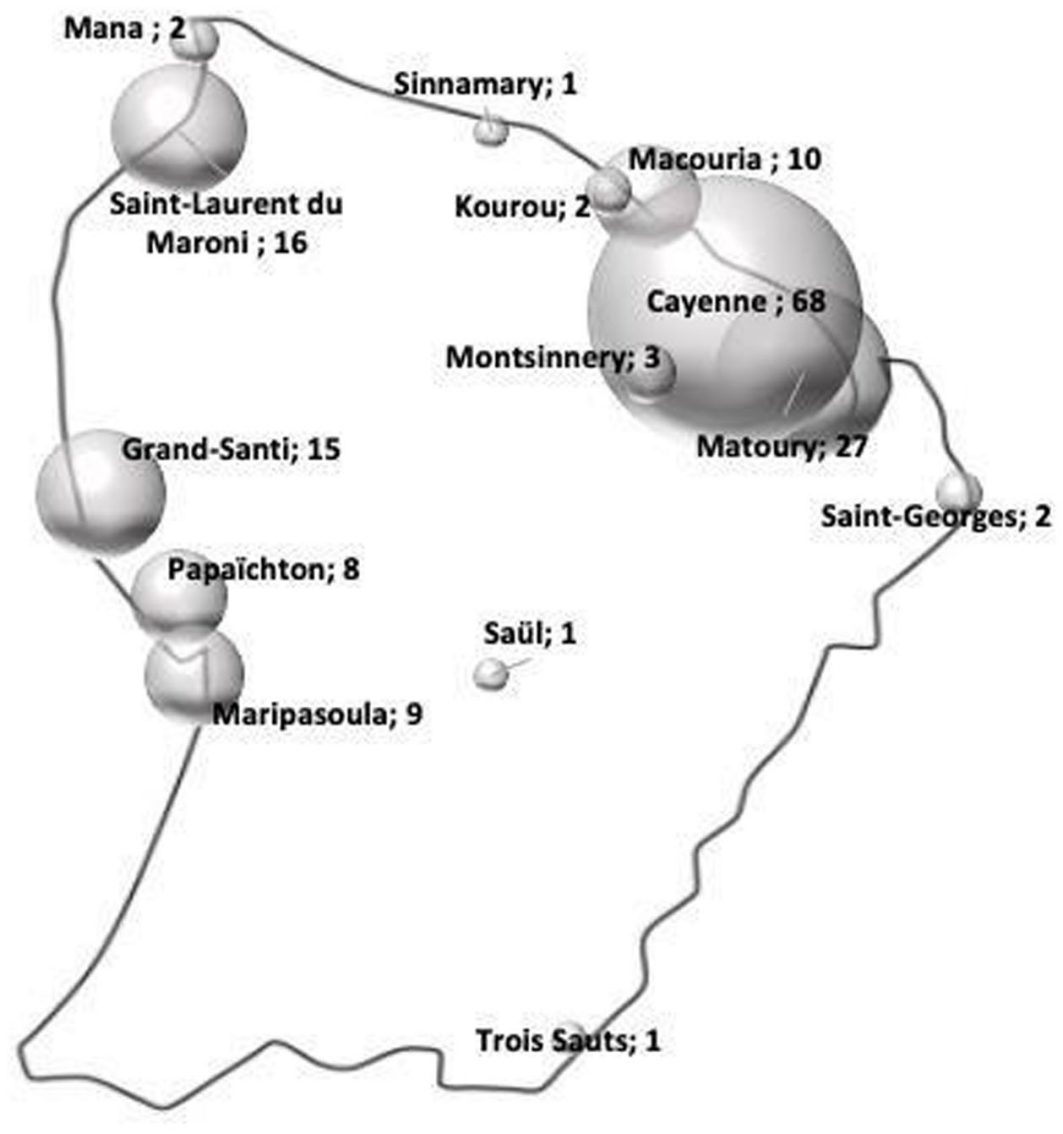
Geographic origin of patients included in the study.

The mean length of hospital stay was 9.8 days. In addition, 10% of patients required medical evacuation to a specialized burn center (see Figure 1).

### Infection and colonization

Skin swab cultures were performed in 102 of the 165 patients. In cases where cultures were not performed, burns generally involved less than 10% TBSA, or the patient was rapidly transferred to mainland France (one patient transferred within 24 h due to severe burns).

Blood cultures were mainly performed in patients presenting fever, elevated inflammatory markers, larger burns, or clinical suspicion of invasive infection. Cultures considered clinically incompatible with bloodstream infection were interpreted as contaminants and excluded from the final analysis. Among the patients who underwent skin sampling, 89 (87%) demonstrated superficial wound colonization. A total of 18 different microorganisms were identified. The most frequently isolated bacterium was methicillin-sensitive *Staphylococcus aureus* (MSSA). Details of the microorganisms isolated from skin cultures are presented in Table 2.

**Table 2:** Skin swab culture results (*n* = 102).

Microorganism	*n* (%)
*Staphylococcus aureus* (MSSA)	55 (53.9)
*Staphylococcus aureus* (MRSA)	5 (4.9)
*Pseudomonas aeruginosa*	8 (7.8)
*Escherichia coli*	6 (5.9)
*Klebsiella* spp.	4 (3.9)
*Enterobacter* spp.	3 (2.9)
*Proteus* spp.	2 (2.0)
*Streptococcus pyogenes*	4 (3.9)
*Enterococcus* spp.	3 (2.9)
*Candida* spp.	2 (2.0)
Other bacteria	10 (9.8)

Blood cultures were performed in 79 patients, mainly in cases of high fever or elevated C-reactive protein levels. Eighteen of 79 blood cultures (22.8%) were positive. Detailed results are presented in Table 3.

**Table 3:** Blood culture results (*n* = 79).

Microorganism	*n* (%)
*Staphylococcus aureus* (MSSA)	6 (7.6)
*Staphylococcus aureus* (MRSA)	1 (1.3)
*Escherichia coli*	3 (3.8)
*Pseudomonas aeruginosa*	2 (2.5)
*Klebsiella* spp.	1 (1.3)
*Enterobacter* spp.	1 (1.3)
*Proteus* spp.	1 (1.3)
*Streptococcus pyogenes*	1 (1.3)
*Enterococcus* spp.	1 (1.3)
Polymicrobial samples	2 (2.5)
Negative cultures	61 (77.2)

Among patients with positive blood cultures and/or wound colonization, 13 patients (7.9%) developed signs of invasive infection, including 12 cases of sepsis and one case of septic shock.

### Antibiotic treatment

Antibiotic therapy was occasionally prescribed empirically in clinically severe situations or in patients considered at high risk of infection despite the absence of confirmed invasive infection. A total of 48 patients (29%) received antibiotic therapy, with a mean treatment duration of 7.3 days (median 7 days; range 1–21 days). Among the 48 patients receiving antibiotics, 11 patients (22.9%) received empirical antimicrobial therapy despite presenting only superficial wound colonization without positive blood cultures or clinical signs of invasive infection.

The most frequently prescribed antibiotic was amoxicillin-clavulanic acid, which was administered in 64.5% of treated patients.

Most patients receiving antibiotics had either positive blood cultures, clinical signs of invasive infection, or burns involving high-risk areas such as the face or extremities.

However, 11 patients received antibiotics despite presenting only wound colonization without positive blood cultures or clinical signs of invasive infection. Although most burns involved less than 10% TBSA, a small subset of patients presented extensive burns exceeding 20% TBSA, including three patients with burns involving more than 40% TBSA.

### Associated factors for invasive infection

Patients presenting signs of invasive infection (sepsis or septic shock) were compared with those without infection. Results of the univariate and multivariate analyses are presented in Table 4.

**Table 4:** Univariate analysis: factors associated with sepsis.

Variable	No sepsis (*n* = 152)	Sepsis (*n* = 13)	*p*-value
Age, months (mean ± SD)	47.6 ± 47.4	21.0 ± 18.5	<0.001
Length of stay, days	8.84 ± 10.1	21.3 ± 14.6	<0.01
TBSA burned (%)	8.3 ± 8.6	14.7 ± 9.8	0.039
High-risk burn areas	65 (43%)	5 (38%)	0.76
Wound colonization	77 (51%)	11 (85%)	0.018
Positive blood culture	5 (3.3%)	8 (62%)	<0.001
Residence outside Cayenne	47 (31%)	9 (69%)	0.011
Central venous catheter	15 (9.9%)	10 (77%)	<0.001
Urinary catheter	16 (11%)	7 (54%)	<0.001
Male sex	89 (59%)	5 (38%)	0.16
Medical evacuation	14 (9.2%)	3 (23%)	0.14

TBSA, Total burned body surface area.

Age was significantly associated with invasive infection. Patients in the sepsis group were younger, with a mean age of 21 months, compared with approximately 4 years in the non-sepsis group.

Patients with invasive infection also had a longer hospital stay (mean 21.3 days vs. 8.84 days).

Other statistically significant factors observed in the sepsis group included:

- A higher rate of positive blood cultures
- A greater use of invasive devices, particularly urinary catheters.

## Discussion

This study provides one of the first descriptions specifically focusing on the epidemiological, microbiological, and infectious characteristics of children hospitalized for burn injuries in French Guiana.

Several important findings emerge from this analysis.

First, the demographic profile observed in our cohort is consistent with that reported in many pediatric burn studies worldwide (1, 2, 6). Most patients were very young, with a median age of 21 months, and boys were slightly overrepresented. These findings reflect the well-known vulnerability of young children to burn injuries, particularly during the first years of life when motor development, curiosity, and limited risk awareness increase the likelihood of domestic accidents. Previous epidemiological studies conducted in Europe and Asia have similarly reported that the majority of pediatric burn injuries occur in children under 5 years of age (7, 8). The interpretation of our findings should take into account the organizational role of Cayenne Hospital Center within the regional healthcare network. The hospital functions both as a referral center for pediatric burn patients across French Guiana and as a transfer point to specialized burn centers in mainland France for the most severe cases. Consequently, the epidemiological profile observed in this cohort may primarily reflect the population managed at this institution rather than the overall burden of pediatric burns across the territory.

Time to first medical contact may represent a clinically relevant parameter in the context of infectious complications. Although most patients in our cohort received medical evaluation within the first 24 h after injury, this variable was not specifically analyzed in relation to infection outcomes because of the retrospective design and limited sample size. Future prospective studies could further investigate the relationship between delayed access to care, referral pathways, and invasive infection risk. However, hospitalization duration may not fully reflect the complete burn management trajectory.

Thermal burns represented the vast majority of injuries in our study, accounting for approximately 80% of cases. Scald injuries caused by hot liquids were the most common mechanism. This pattern is consistent with previously published studies and reflects the predominance of domestic accidents involving hot liquids during cooking or food preparation. Young children are particularly exposed to this type of injury due to their proximity to caregivers during household activities and their limited ability to recognize danger (9).

The geographic distribution of patients highlights two main population clusters: the Cayenne metropolitan area and remote communities located along the Maroni River near the Surinamese border. Despite the geographic isolation of many communes in French Guiana, the time to first medical contact was relatively short, with most patients receiving medical evaluation within 24 h after the burn injury. This finding likely reflects the presence of a network of decentralized healthcare centers throughout the territory, which facilitates early access to primary care even in remote areas.

However, the mean length of hospital stay in our cohort was relatively long compared with that reported in some other studies. Length of stay should also be interpreted cautiously, as some patients were transferred after initial stabilization while others were admitted after prior management in remote healthcare centers. This bidirectional patient flow may have influenced hospitalization duration.

Although most patients in our cohort received medical evaluation within the first 24 h after injury, this parameter was not specifically analyzed in relation to infectious outcomes due to the limited sample size and retrospective design of the study. We highlighted that future prospective studies could further explore the relationship between delayed access to care, referral pathways, and the risk of invasive infection, particularly among children originating from remote areas of French Guiana.”

Children originating from remote communes may face more complex referral pathways and differences in access to specialized burn management, which could contribute to increased infectious complications. Interestingly, although time to first medical contact was relatively short even among patients from remote areas, children originating from isolated communes presented a higher frequency of invasive infection. This observation may reflect differences in access to specialized care, referral pathways, transport conditions, or continuity of wound management. Further prospective studies would be valuable to better understand these mechanisms.

Wound colonization was common in our cohort, affecting the majority of patients who underwent microbiological sampling. The relatively high blood culture positivity rate may partly reflect selective sampling practices targeting clinically severe or febrile patients. As expected, methicillin-sensitive *Staphylococcus aureus* was the most frequently isolated microorganism from both wound and blood cultures. This finding is consistent with previous studies showing (10, 11). Despite the high rate of wound colonization, the proportion of patients who developed invasive infection remained relatively low. This finding suggests that colonization does not necessarily progress to systemic infection in most cases, provided that appropriate wound care and clinical monitoring are implemented.

Approximately one-quarter of antibiotic prescriptions in our cohort may have represented potentially avoidable empirical therapy. The interpretation of antibiotic prescribing practices should be approached cautiously. Although difficulty distinguishing systemic inflammatory response syndrome from early sepsis (12, 13) may partly explain empirical antibiotic use, other contributing factors such as clinical uncertainty, local prescribing habits, and burn severity may also have played a role. In addition, the frequent use of amoxicillin-clavulanic acid likely reflects local pediatric prescribing practices and the absence of a dedicated pediatric burn unit with standardized antimicrobial stewardship protocols. This observation highlights the importance of antimicrobial stewardship strategies and standardized diagnostic criteria in pediatric burn care. Although difficulty distinguishing SIRS from sepsis may partly explain empirical antibiotic use, other contributing factors such as clinical uncertainty, local prescribing practices, and burn severity likely also contributed. The frequent use of amoxicillin-clavulanic acid likely reflects local pediatric prescribing practices and the absence of standardized pediatric burn antimicrobial stewardship protocols.

The identification of associated factors for invasive infection was therefore particularly important. In our study, younger age and a larger total burned body surface area were significantly associated with the occurrence of sepsis. These findings are consistent with previous reports showing that extensive burns and young age are major predictors of infectious complications in pediatric burn patients. Younger children may be particularly vulnerable due to their immature immune system and thinner skin barrier (14, 15).

In addition, the presence of invasive medical devices, particularly urinary catheters and central venous catheters, was associated with a higher risk of infection. This observation is consistent with the well-established role of invasive devices as potential sources of nosocomial infection in critically ill patients.

From a clinical perspective, these results emphasize the importance of early identification of patients at higher risk of infection (16). Younger children, patients with larger burn surface areas, and those requiring invasive devices should be closely monitored for early signs of sepsis (14, 15). Preventive strategies should focus on meticulous wound care, strict infection control measures, and rational antibiotic use (16). Limiting unnecessary antibiotic prescriptions is particularly important in order to reduce the emergence of antimicrobial resistance.

Interestingly, from a preventive perspective, French Guiana could draw on several child burn prevention programs that have already been implemented and evaluated in various countries (17–19).

This study has several limitations that should be acknowledged. First, the retrospective design may have introduced information bias related to incomplete or heterogeneous medical records. Second, microbiological sampling was not systematically performed in all patients, particularly those with small burn surface areas, which may have influenced the observed rates of colonization and infection. Time to first medical contact and burn depth were not sufficiently documented to allow robust analytical exploration. Selective microbiological sampling may have overestimated colonization and bloodstream infection rates. Detailed surgical management data were inconsistently documented in the medical records and could not be reliably analyzed. Finally, this study was conducted in a single center, which may limit the generalizability of the findings to other healthcare settings.

Despite these limitations, our study includes a relatively large number of pediatric patients and covers a 6-year period, providing valuable insight into burn epidemiology in a region where data remain scarce. To our knowledge, this is one of the first studies specifically focusing on infectious complications of pediatric burns in French Guiana.

## Conclusion

The findings of this single-center retrospective cohort suggest that invasive infection remains relatively uncommon among hospitalized pediatric burn patients despite frequent wound colonization. Younger age, larger burn surface area, residence in remote areas, and the use of invasive devices were associated with invasive infection. These findings should be interpreted cautiously given the retrospective design and the single-center setting of the study. Future prospective studies would be valuable to better characterize the associated factors for infection and to evaluate the impact of standardized protocols for burn management and antibiotic stewardship in this population.

## Data Availability

The raw data supporting the conclusions of this article will be made available by the authors, without undue reservation.
